# Efficiency Enhancement Technology of *Dastarcus helophoroides* (Coleoptera: Bothrideridae) for Controlling *Monochamus alternatus* (Coleoptera: Cerambycidae): Drilling Optimization and Biological Collaboration

**DOI:** 10.3390/insects16111138

**Published:** 2025-11-07

**Authors:** Jiale Li, Min Zhang, Zhilan Li, Xiaohui Li, Yong Peng, Wenxiu Zhou, Zhengping Zhao, Xuewu Yan

**Affiliations:** 1 Hunan Academy of Forestry, Changsha 410004, China; ljl670509@163.com (J.L.); zhangmin123@hnlky.cn (M.Z.); 2 Hunan Biodiversity Conservation Center, Changsha 410004, China; julie_0819@126.com; 3 Forestry Research Institute of Shangrao City, Shangrao 334000, China; srlksxb@126.com (X.L.); pengyong1102@sina.com (Y.P.); wenxiuzhou002@gmail.com (W.Z.)

**Keywords:** pine sawyer beetle, pine wilt disease, parasitic natural enemy, biological synergy, release technology, pest management

## Abstract

*Dastarcus helophoroides* Fairmaire, a parasitic natural enemy widely used in the biological control of *Monochamus alternatus* Hope, possesses strong abilities to search for hosts and prey. To enhance the biological control efficacy of *D. helophoroides* against *M. alternatus*, this study systematically evaluated the effectiveness of enhancing control through methods such as drilling holes in lure logs and introducing *D. helophoroides* carrying *Pyemotes zhonghuajia* or *Beauveria bassiana* under controlled indoor conditions. The results showed that: (1) Releasing *D. helophoroides* eggs (after 30 days) and releasing adults (after 120 days) were both effective in controlling *M. alternatus*, with the best control effects achieved when 50 eggs/m and 3 adults/m were released. The release of adults took longer to show effects but had better results than the release of eggs in the later stages. (2) The location and number of holes drilled in lure logs significantly influenced the efficiency of *D. helophoroides* in searching for and parasitizing *M. alternatus*, while the diameter of the holes was not significant. Considering the highest control efficacy and minimizing damage to the tree, the optimal conditions were releasing *D. helophoroides* adults 2 cm above *M. alternatus* fecal holes, with 0.4 cm in diameter and 2 holes/m release density, which significantly promoted the invasion of *D. helophoroides* adults into the borer tunnels and increased the *M. alternatus* mortality rate. (3) Releasing *D. helophoroides* adults carrying *P. zhonghuajia* or *B. bassiana* significantly improves the control efficacy against *M. alternatus*. The higher the number of *D. helophoroides* adults released per log segment and the greater the quantity of *P. zhonghuajia* or *B. bassiana* that they carry, the higher the mortality rate of *M. alternatus*. After 60 days of release, the highest mortality rate of *M. alternatus* was observed in treatments where *D. helophoroides* (released at densities of 3 adults per meter of log segment) carried *P. zhonghuajia* (0.6 million/m) or were sprayed with *B. bassiana* (3 g/m). The synergistic effect of carrying *P. zhonghuajia* was evident, but the overall control efficacy of spraying *B. bassiana* was limited. In summary, optimizing drilling techniques and combining biological release strategies under laboratory conditions can significantly enhance the control efficacy against *M. alternatus*, providing data support for *D. helophoroides* future application in forest pest control.

## Simple Summary

*Monochamus alternatus* is a highly destructive wood-boring pest in Chinese coniferous forests and the primary vector of pine wilt disease caused by *Bursaphelenchus xylophilus*. Its control is challenging due to the complex damage mechanism and coevolution with the pine wood nematode *B. xylophilus*. *Dastarcus helophoroides*, a key natural enemy of *M. alternatus*, exhibits strong potential for biocontrol but requires improved release techniques and synergy strategies. This study systematically demonstrates that optimizing drill hole parameters (location, number, and diameter) in lure logs and using *D. helophoroides* carrying *Pyemotes zhonghuajia* or *Beauveria bassiana* significantly enhances host search and parasitism efficiency. These findings provide technical support for integrated management of *M. alternatus* and pine wilt disease in subsequent practical forest applications.

## 1. Introduction

*Monochamus alternatus* Hope (Coleoptera: Cerambycidae) is one of the most destructive wood-boring pests in East Asia, primarily targeting pine plants such as *Pinus massoniana*, *P. armandii*, *P. tabulaeformis*, etc. [1]. Its larvae feed on the phloem and xylem of the host, forming crisscrossing galleries that severely damage the tree’s conducting tissue [2]; after emergence, adults feed on healthy pine branches to supplement nutrients and lay eggs [3]. Additionally, *M. alternatus* serves as the primary vector insect for the pine wood nematode (*Bursaphelenchus xylophilus*, PWN), a significant invasive pest in Chinese forestry, capable of transmitting pine wilt disease (PWD), which inflicts devastating impacts on Chinese pine forest ecosystems. While many insect species can carry PWN, only *Monochamus* serve as vector insects for *B. xylophilus* [4,5]. The life cycles of *Monochamus* are consistent with that of *B. xylophilus*, and the wounds caused by its supplementary nutrition and oviposition behavior provide entry points for *B. xylophilus* to infect new plants [6]. Since it invaded Nanjing City in 1982, PWD is the most severe forest disease in China in recent decades, and it has rapidly spread to 18 provinces (https://www.forestry.gov.cn/c/www/gsgg/612537.jhtml, accessed on 4 November 2025) causing catastrophic economic and ecological losses [7,8]. In 2020, China lost $7.4 billion due to PWD, including $1.11 billion in direct economic loss and $6.29 billion in ecological service value [9]. The chemical ecology of *M. alternatus* is complex, with its host location, mating, and egg-laying regulated by plant volatiles (such as α-pinene) and sex pheromones, and it has a coevolutionary relationship with *B. xylophilus* [10,11,12], further complicating its control.

Current control strategies for *M. alternatus* include chemical agents, physical traps, and biological control [13,14]. However, due to the concealed nature of *M. alternatus* galleries, chemical pesticides (such as Emamectin, and Avermecti) struggle to penetrate effectively. Additionally, overuse can harm natural enemies and other beneficial insects (such as honeybees), leading to environmental pollution, increased pest resistance, and excessive pesticide residues in forest products [15,16,17]. While plant volatiles and pheromone traps are targeted, they have short residual efficacy and are primarily used for monitoring and controlling *M. alternatus* adult populations, and factors such as natural environmental and host plant differences can affect the effectiveness of attractants [12,18,19,20]. Therefore, environmentally friendly, sustainable, and efficient biological control strategies have become an urgent need in contemporary biological control research. Biological control of *M. alternatus* primarily relies on natural enemies (parasitic and predatory insects) and microorganisms, such as *Dastarcus helophoroides*, *Scleroderma guani*, and *Beauveria bassiana* [21,22]. Among them, *D. helophoroides* stands out as the most effective natural enemy insect due to its efficient parasitic behavior and longevity [23,24].

*Dastarcus helophoroides* Fairmaire (Coleoptera: Bothrideridae) demonstrates significant potential for controlling wood-boring pests such as *M. alternatus*, *Massicus raddei* (Coleoptera: Cerambycidae), and *Anoplophora glabripennis* (Coleoptera: Cerambycidae), etc., due to its strong host-seeking ability and efficient parasitic predation behavior [14,25,26,27]. As a result, it has been widely applied in the biological control of the pine wood-boring beetle both domestically and internationally in recent years. However, there are still some bottlenecks that need to be broken through in the research on the control of *M. alternatus*, such as insufficient investigation into parameters (diameter, location, and number of drill holes on the lure logs), as well as the release method, quantity, and timing of *D. helophoroides*, and insufficient synergistic enhancement capabilities [28]. Hence, optimizing the drilling and release techniques for lure logs and exploring synergistic mechanisms with other biological factors are key research directions for enhancing the effectiveness of *D. helophoroides* control against *M. alternatus* in the future [29,30].

This study systematically evaluated the long-term efficacy of *D. helophoroides* released at different developmental stages and densities under controlled conditions, optimized lure log drilling strategies to enhance host search and invasion efficacy, and explored synergistic interactions and optimal ratios between *D. helophoroides* adults and the biocontrol agents *Pyemotes zhonghuajia* (Acari: Pyemotidae) and *Beauveria bassiana* (Hypocreales: Cordycipitaceae). The purpose of this study was to enhance the control efficacy of *D. helophoroides* against *M. alternatus* (and PWD it transmits), provide a theoretical foundation for developing composite biological control technologies based on *D. helophoroides*, and offer technical parameter support for subsequent practical applications in forest settings, including drilling holes in lure logs and releasing *D. helophoroides*.

## 2. Materials and Methods

### 2.1. Treatment Materials

*Dastarcus helophoroides* adults were reared in the Southern Natural Enemies Breeding and Application Engineering Technology Center using artificial feed in indoor conditions; all *D. helophoroides* eggs were laid by adults reared indoors. *Pyemotes zhonghuajia* was provided by the Changli Fruit Tree Research Institute of the Hebei Academy of Agricultural and Forestry Sciences and expanded at the Southern Natural Enemy Breeding and Application Engineering Technology Research Center. *Beauveria bassiana* was provided by Jiangxi Tianren Ecology Co., Ltd. (Ji’an, China).

The lure logs for *M. alternatus* were set up in a *P. massoniana* forest within the Jiangya Forest Farm in Cili County, Hunan Province. After being felled in September of the same year, the lure logs (all newly cut fallen and obtained from dying or newly dead wood with a diameter range of 10–20 cm) were transported to the Hunan Province Natural Enemy Breeding Center for storage. The lure logs were then cut into 1-m-long segments, and those with 5~8 distinct *M. alternatus* fecal holes were selected for use.

### 2.2. Separate Release Treatment of Dastarcus helophoroides Eggs and Adults

Egg control: 2850 healthy eggs were selected and released at density gradients of 10, 20, 30, 50, and 80 eggs/m onto lure logs. Each treatment was set with 5 lure logs and repeated 3 times. The lure logs were cultured in an artificial climate chamber at 25 °C, 80% humidity, and approx. 12L:12D photoperiod.

Adult control: 225 healthy adults of similar size were selected and released at density gradients of 1, 2, 3, 4, and 5 adults/m onto lure logs, and were set the number of lure logs as described above and cultured under the same conditions.

Finally, eggs were surveyed 30 days after release, and adults were surveyed 60 and 120 days after release, which was related to the developmental cycle of *D. helophoroides* [28]. The mortality rate of *M. alternatus* within each lure log was calculated, and the control efficacy of releasing *D. helophoroides* separately was observed under different insect stages and densities. An untreated control group (lure logs with no release of *D. helophoroides*) was included to assess natural mortality under indoor conditions. The mortality rates reported in figures and tables have been corrected by subtracting the natural mortality rate observed in the control group.

### 2.3. Release Treatment of Dastarcus helophoroides Adults After Drilling Holes in Lure Logs

Drilling location and diameter test: 175 lure logs were randomly selected and placed in fine-mesh insect rearing cages to prevent escape of *D. helophoroides* adults. And then, the 175 logs were randomly assigned to 35 treatment combinations (7 locations × 5 diameters), with 5 replicates per combination. The cages were then placed in an artificial climate chamber (temperature 28 ± 1 °C, humidity 75 ± 5%). Drill holes (depth 2.5 cm) in the lure logs were used by drill bits with diameters of 0.2, 0.4, 0.6, 0.8, and 1.0 cm. The number of drill holes were matched to the number of fecal holes in the lure logs. Seven drill hole location treatment groups were established: ① directly above the fecal hole; ② 1 cm above the fecal hole; ③ 2 cm above the fecal hole; ④ 1 cm below the fecal hole; ⑤ 2 cm below the fecal hole; ⑥ random drilling away from the fecal hole; ⑦ without drilling (control), and each treatment group was repeated 5 times. After drilling, 5 adults were released onto the surface of each lure log. After 30 days of release, the bark was stripped off, and the lure logs were carefully split with an axe. The actual number of surviving *D. helophoroides* adults, the number entering the galleries, and the number of *M. alternatus* parasitized and predated on each lure log were counted, and the mortality rate of *M. alternatus* was calculated.

Drilling hole number test: 75 lure logs were randomly selected, and the optimal drilling method selected above was used to set up three drilling hole number treatments: 1, 2, and 3 holes/m, with 25 lure logs per group. After drilling, we released one pair (1 male and 1 female) of *D. helophoroides* adults into each lure log. After 150 days of release, the same method was used to dissect the lure logs, and then we counted the number of *M. alternatus* inside the lure logs, as well as the number of *M. alternatus* parasitized or preyed upon, and calculated the mortality rate of *M. alternatus*.

All *D. helophoroides* adults were released directly onto the surface of each lure log without additional attractants. Host location and drill hole entry were achieved primarily through random crawling and antennal sensing.

### 2.4. Release Treatment of Dastarcus helophoroides Adults Carrying Synergistic Species

*Dastarcus helophoroides* adults + *P. zhonghuajia* group: the release density was set as 1, 2, or 3 adults/m, corresponding to *P. zhonghuaji* release quantities of 0.2, 0.4, or 0.6 million/m. A control group was also established with the same release quantities of *P. zhonghuaji* (0.2, 0.4, and 0.6 million/m). We chose healthy and lively *D. helophoroides* adults, and directly let them crawl over a culture dish containing newly hatched *P. zhonghuajia* for 5 min. *Pyemotes zhonghuajia* climbed on the body surface of *D. helophoroides* adults (mainly at the folds of the thorax and basal ganglia). The exposure time of 5 min was selected based on preliminary trials without causing beetle stress.

*Dastarcus helophoroides* adults + *B. bassiana* group: the release density was set as 1, 2, or 3 adults/m, corresponding to *B. bassiana* (spore suspension) application rates of 1, 2, or 3 g/m. A control group was also established with the same application rates of *B. bassiana* (1, 2, or 3 g/m), “g/m” refers to the gram of *B. bassiana* spore powder used per meter of wood section. For the treatment group, healthy and active *D. helophoroides* adults were allowed to crawl directly over sporulating cultures of *B. bassiana* for approximately five minutes. Subsequently, individuals that survived and remained active were selected and released onto the lure logs at the designated densities. All other procedures were consistent with the control group. Parasitism was confirmed by visual inspection of *D. helophoroides* attached to the host *M. alternatus* larvae or pupa. *Beauveria bassiana* infection was verified by the presence of fungal mycelia on the host body and microscopic identification of spore.

In the above two groups, each group was applied to 20 lure logs, repeated 3 times, and surveyed 60 days after release. The number of *M. alternatus* and the number of those parasitized and predated were counted in each lure log, and the mortality rate was calculated.

### 2.5. Statistical Analysis

All data were preliminarily processed using Excel, and one-way analysis of variance (ANOVA) and Duncan’s multiple comparisons were performed using SPSS 27.0. The minimum significant difference at a 95% probability was used as the post hoc test, and Hiplot online graphing website was used for graphing.

## 3. Results

### 3.1. Control Efficacy of Releasing Dastarcus helophoroides Separately Under Different Developmental Stages and Densities

#### 3.1.1. Effect of Releasing Eggs

**Figure 1:**
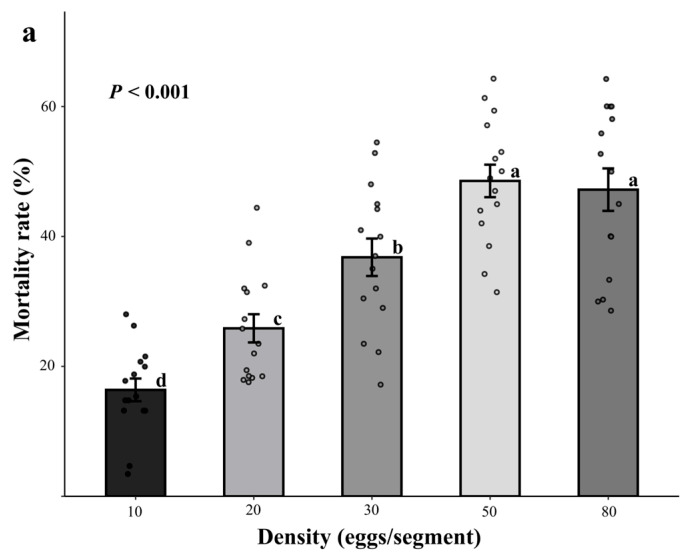

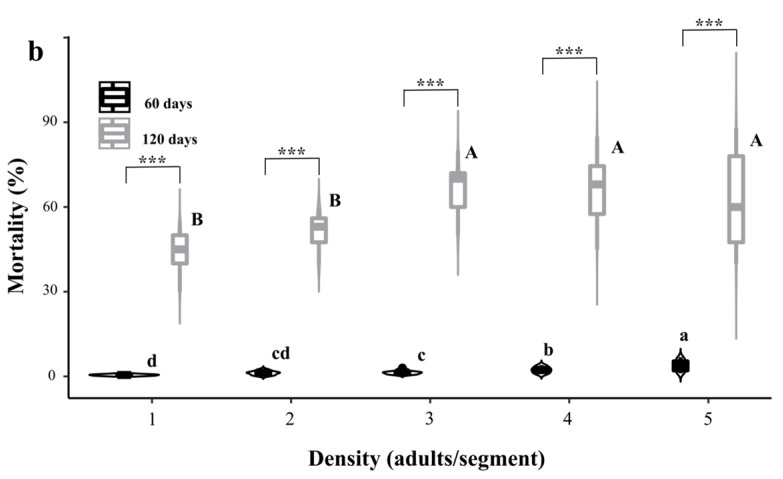
Effectiveness of releasing *Dastarcus helophoroides* eggs and adults at different densities on the control of *Monochamus alternatus*. (**a**) Different lowercase letters following the data indicate significant differences in *M. alternatus* mortality rates at different release densities 30 days after releasing *D. helophoroides* eggs (*p* < 0.05); (**b**) Different lowercase letters and uppercase letters above the bars indicate significant differences in the mortality rate of *M. alternatus* at different release densities 60 days and 120 days after releasing *D. helophoroides* adults, respectively (*p* < 0.05), and the asterisk (***) indicates the significant difference compared the two time points across all densities (*p* < 0.001).

Different release densities significantly affected the efficacy of *D. helophoroides* eggs in controlling *M. alternatus* (*p* < 0.001), and as the release quantity increased, the mortality rate of *M. alternatus* gradually increased (Figure 1a). After 30 days of release, the mortality rates for the 10, 20, 30, 50, and 80 eggs/m treatment groups were 16.4 ± 6.7%, 25.8 ± 8.5%, 36.7 ± 11.3%, 48.5 ± 9.5%, and 47.2 ± 12.7%, respectively. The mortality rate was highest in the 50 eggs/m group, followed by the 80 eggs/m group, with no significant difference between the two groups, but both were significantly higher than the other three groups (*p* < 0.05).

#### 3.1.2. Effect of Releasing Adults

Regarding the increase in release days, the mortality of *M*. *alternatus* was significantly different between 60 and 120 days after release (*p* < 0.001). Different release densities significantly influenced the effect of *D*. *helophoroides* adults on the control of *M*. *alternatus* (*p* < 0.001), with the control effect gradually improving as the release quantity of adults increased (Figure 1b).

After 60 days of release, the mortality rates of *M*. *alternatus* in all treatment groups (1~5 adults/m) were relatively low, with mortality rates of 0.5 ± 0.3%, 1.2 ± 0.7%, 1.4 ± 0.7%, 2.3 ± 1.0%, and 3.8 ± 1.9%, respectively. The mortality rate in the group with 5 adults/m was significantly higher than that in the other groups (*p* < 0.05).

After 120 days of release, the mortality rate of *M*. *alternatus* significantly increased, with mortality rates of 43.7 ± 7.2%, 51.2 ± 7.1%, 65.8 ± 9.5%, 66.5 ± 12.4%, and 63.4 ± 16.9% for the treatment groups (1~5 adults/m), respectively. The mortality rates for 3~5 adults/m were significantly higher than those for 1~2 adults/m (*p* < 0.05). However, there were no significant differences among the 3, 4, and 5 adults/m groups, nor between the 1 and 2 adults/m groups.

### 3.2. Control Efficacy of Drill Hole on Dastarcus helophoroides Searching and Parasitic Behavior

#### 3.2.1. Effect of Lure Logs Drill Hole Location and Diameter

**Figure 2:**
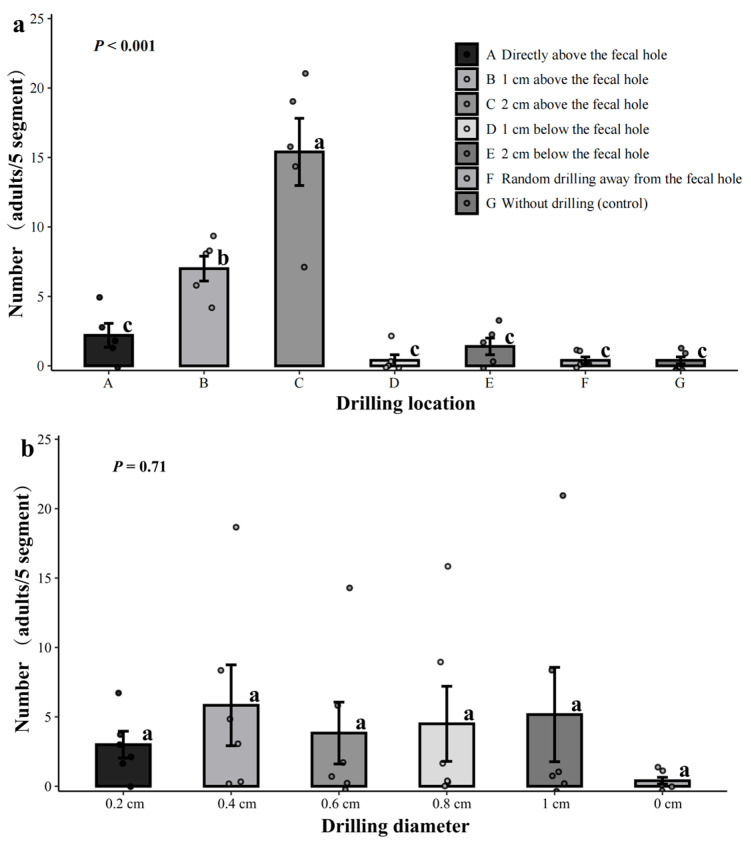
Effect of lure logs drilling location and diameter on the entry of *Dastarcus helophoroides* adults into *Monochamus alternatus* galleries. (**a**) Different lowercase letters following the data indicate significant differences in the number of *D. helophoroides* adults entering *M. alternatus* galleries at different drilling locations 30 days after release (*p* < 0.05); (**b**) There was no significant difference in the number of *D. helophoroides* adults entering *M. alternatus* galleries at different drilling diameters. Note: 0 cm refers to the no-drilling control group.

Different drill hole locations significantly affected the number of *D*. *helophoroides* adults entering *M*. *alternatus* galleries (*p* < 0.001). The highest number of *D*. *helophoroides* adults was observed 2 cm above the fecal hole, and this was significantly higher than other locations (*p* < 0.05) (Figure 2a). The number of *D*. *helophoroides* entering the galleries was as follows: 2 cm above the fecal hole (77 adults) > 1 cm above the fecal hole (35 adults) > directly above the fecal hole (10 adults) > 2 cm below the fecal hole (7 adults) > 1 cm below the fecal hole (2 adults) = randomly drilled holes away from the fecal hole (2 adults) = no-drill control group (2 adults).

There was no significant difference in the number of *D*. *helophoroides* adults entering *M*. *alternatus* galleries under different drilling diameters (0.2 cm~1 cm, even including no-drill control, i.e., 0 cm) (*p* = 0.71) (Figure 2b). Among these, when drilling at 2 cm above the fecal hole, the treatment groups with a hole diameter of 0.4 cm (19 adults) and 1.0 cm (21 adults) had the highest total number of *D*. *helophoroides* entry. Although diameter alone did not significantly affect entry rate, a significant interaction between location and diameter was observed, indicating that diameter becomes relevant when the drilling location is optimal.

The greater the number of *D*. *helophoroides* adults in the infested galleries, the greater the number of *M*. *alternatus* they parasitize and prey upon (Table S1). Additionally, the drilling location significantly influences the lethal effect of *D*. *helophoroides* adults on *M*. *alternatus* (*p* < 0.001). Correspondingly, when drilling holes 2 cm above the fecal holes, the total parasitic predation and mortality rates of *M*. *alternatus* were the highest, significantly higher than those of the other drilling location treatment groups (*p* < 0.05); When drilling holes 2 cm above the fecal holes, there were differences in mortality rates among treatments with different drill hole diameters (0.2~1.0 cm). Among the groups with diameters of 0.4 cm, 0.6 cm, 0.8 cm, and 1.0 cm, the differences were not significant, but all were significantly higher than the 0.2 cm diameter group (*p* < 0.05). Considering the highest pest control efficacy and minimizing damage to the tree, subsequent experiments selected drilling at a location 2 cm above the fecal hole with a diameter of 0.4 cm.

#### 3.2.2. Effect of Lure Logs Drill Hole Number

Based on the optimal drilling method selected above, which involves drilling holes 2 cm above the fecal holes with a diameter of 0.4 cm, the number of holes drilled per log significantly affects the mortality rate of *M*. *alternatus* (*p* < 0.05). There was no significant difference in mortality rates between the 2 holes/m and 3 holes/m groups, but both were significantly higher than the 1 hole/m group (Table 1). Specifically, after 150 days of release, *D*. *helophoroides* adults could successfully reproduce one generation in the lure logs. Therefore, in the lure logs where *D*. *helophoroides* adults were released in the 1, 2, and 3 holes/m groups, the total number of *D*. *helophoroides* larvae and adults was 56, 67, and 53, respectively, while the total number of *M*. *alternatus* was 157, 162, and 154, respectively. Among these, the 1 hole/m group had 41 parasitized individuals and 4 preyed individuals, with a mortality rate of 28.6 ± 2.8%; the 2 holes/m group had 54 parasitized individuals and 4 preyed individuals, with a mortality rate of 35.8 ± 2.3%; the 3 holes/m group had 50 parasitized individuals and 6 preyed individuals, with a mortality rate of 36.4 ± 1.0%. Therefore, considering both labor costs and work efficiency, it was recommended to drill 2 holes/m for field applications.

### 3.3. Synergistic Control Efficacy of Dastarcus helophoroides

#### 3.3.1. Effect of Carrying *Pyemotes zhonghuajia*

Releasing *D. helophoroides* adults carrying *P. zhonghuajia* significantly enhanced the control efficacy against *M. alternatus* (*p* < 0.001), and the mortality rate of *M. alternatus* increased with the release density of *D. helophoroides* adults and *P. zhonghuajia* (Table 2).

Specifically, the mortality rates of *M. alternatus* were 29.7 ± 1.8%, 48.2 ± 2.7%, and 67.6 ± 1.1% when 1, 2, and 3 adults/m were released, respectively (*p* < 0.05); Additionally, the mortality rate of *M. alternatus* was significantly higher than that of the sole *P. zhonghuajia* treatment group under the same release density (*p* < 0.05). In the sole release of the *P. zhonghuajia* group (0.2, 0.4, and 0.6 million individuals/m), the rates were 15.5 ± 1.4%, 24.5 ± 2.7%, and 30.4 ± 1.0%, respectively.

#### 3.3.2. Effect of Carrying *Beauveria bassiana*

The release of *D. helophoroides* adults sprayed with *B. bassiana* resulted in a statistically significant increase in the mortality of *M. alternatus* (*p* < 0.05). Although a positive dose–response relationship was observed, where mortality increased with the number of treated adults and the amount of *B. bassiana* applied per log (Table 3), the overall mortality rate remained low in absolute terms.

In the group where *D. helophoroides* adults were sprayed with *B*. *bassiana*, there was no significant difference in the mortality rate of *M. alternatus*. The mortality rates of *M. alternatus* were 12.0 ± 1.5%, 11.1 ± 2.5%, and 15.2 ± 1.3% for 1, 2, and 3 adults/m, respectively. However, the mortality rate of *M. alternatus* was significantly higher than that of the group treated with *B*. *bassiana* alone at the same application rate (*p* < 0.05). In the groups treated with *B*. *bassiana* alone (1, 2, and 3 g/m), the mortality rates of *M. alternatus* were 5.2 ± 1.3%, 7.1 ± 2.4%, and 8.1 ± 1.6%, respectively.

## 4. Discussion

The release form (eggs/adults) and density of *D. helophoroides* significantly influenced the control efficacy against *M. alternatus*. Egg release resulted in rapid suppression, while adult release exhibited delayed but superior long-term efficacy. Some studies also believed that the control effect of the release of *D. helophoroides* adults was significantly higher than that of eggs [31]. This is closely related to the developmental cycle and search behavior of *D. helophoroides*, and the hatched larvae can directly invade *M. alternatus* galleries and parasitize *M. alternatus* pupae, while *D. helophoroides* adults require a relatively long oviposition preparation period [32,33].

In the group where *D. helophoroides* adults were released, after 60 days, due to the short duration of the experiment, *Dastarcus helophoroides* could only complete one generation of development, resulting in a relatively low overall mortality rate of *M. alternatus*. In indoor experiments, it was also proposed that when the ratio of *D. helophoroides* eggs and adults to fecal holes was 16:1 and 1:1, respectively, the population decline rate of *M. alternatus* was high after 45 and 60 days of release [34], which was due to the use of a higher release density than in our study. After 120 days of release, the mortality of *M. alternatus* increased significantly since it had been continuously bred for several generations in the lure logs, and many of the offspring of adults re-searched for the host *M. alternatus* and parasitized successfully. Additionally, during the experiment, we observed that some *D. helophoroides* adults could parasitize and prey on more than two *M. alternatus*, accounting for approximately 5% of the total released population. In our experimental results, from a long-term perspective, releasing *D. helophoroides* eggs and adults can generally achieve relatively ideal control effects against *M. alternatus*. Future studies would benefit from quantifying egg hatch rates and larval establishment to more directly link release density to the number of parasitic individuals. However, the prolonged effective period of *D. helophoroid* adult release (60~90 days) remains a limitation in addressing explosive pest outbreaks. Future research could explore an “egg + adult” combined release model to balance timeliness and sustainability [32,35].

Drilling technology can enhance its positioning capabilities; the location, diameter and number of holes in the luring wood all affected the ability of *D. helophoroid* adults to search for and enter *M. alternatus* galleries and its control effect on *M. alternatus* to a certain extent. Among these factors, the location and number of drill holes had a significant impact, with the location being the most critical (2 cm above the fecal hole of the *M. alternatus*) in our study. We found that in the experiment, *M. alternatus* had a large appetite and vigorous defecation, and the excreted feces were generally pushed out of the fecal hole through its own movement. These feces were often compressed into tight, elongated shapes during the pushing process, blocking the fecal hole and the space above it. Although *D. helophoroid* adults can consume a portion of *M. alternatus* feces, the amount is minuscule compared to the total volume of *M. alternatus* feces [28]. This may be the key reason limiting the successful entry of *D. helophoroid* into the galleries for predation or parasitism, and also explains the significant differences in experimental outcomes due to varying drilling locations. Since *M. alternatus* have a habit of boring upward, fecal accumulation is minimal above 2 cm in the galleries (though some fecal matter is present, it is loosely accumulated); therefore, once *D. helophoroid* enter *M. alternatus* galleries through the drilled holes, they can quickly initiate parasitic behavior [36,37]. We observed that after 30 days, when dissecting the lure logs, over 90% of *D. helophoroid* adults had entered the drilled holes, but the number of *D. helophoroid* that could enter *M. alternatus* galleries varied greatly among groups, with a few remaining hidden in the bark. Due to the short experimental period, the *D. helophoroid* adult survival rate was good, with only 7 dead adults found among the 175 lure logs. Releasing *D. helophoroid* adults with drilling optimization technology can significantly improve the effect and is suitable for most epidemic areas, especially for medium-to-low-incidence forest stands that require long-term and stable control.

When *D. helophoroid* adults encounter drill holes with a diameter of 0.2 cm, since the holes are narrower than their body, some *D. helophoroid* will chew on the surrounding wood tissue until their entire bodies are fully inserted into drill galleries, and others will abandon and continue searching for other suitable holes [38]. Similarly, our experimental results also showed that when the drill hole diameter was >0.4 cm, *M. alternatus* mortality rate was significantly higher than that of the 0.2 cm hole diameter group. After 150 days, except for the galleries where no *M. alternatus* survived, all *D. helophoroid* adults that entered the galleries were able to prey on or parasitize *M. alternatus*, especially when multiple *D. helophoroid* entered the same gallery, *M. alternatus* were almost completely consumed. The results indicated that the optimal combination was drilling holes 2 cm above the fecal holes, with a hole diameter of 0.4 cm and 2 holes/m, which not only had minimal damage to the trees, but also had better control effect on *M*. *alternatus*. There is an obvious selective mating behavior in *D. helophoroid* adults, and the body size of the parental generation significantly affects the reproductive fitness and offspring development [39]. Therefore, when releasing *D. helophoroid* adults, the impact of size differences in the source population should be considered, and individuals that are larger and of similar size should be used whenever possible.

Insufficient synergistic efficacy can be enhanced by combining other natural enemy insects to improve pest control effectiveness. For example, the combined release of *S*. *guani* and *D. helophoroid* can significantly improve pest control efficacy [40]. However, other studies have proposed that the parasitism rate under the interference of *S*. *alternatusi* is significantly lower than that under the independent effect of *D. helophoroid* [41]. The results of our experiment indicated that *D. helophoroid* adults carrying *P. zhonghuajia* can significantly enhance the pest control efficacy against *M*. *alternatus*. It is worth noting that approximately 80% of *M*. *alternatus* dead individuals in the group carrying *P*. *zhonghuajia* were caused by *P*. *zhonghuajia*, while only approximately 20% were caused by direct parasitism and predation by *D. helophoroid*, indicating that *D. helophoroid* can serve as an effective carrier for *P*. *zhonghuajia*, significantly enhancing the efficiency of *P*. *zhonghuajia* reaching and parasitizing *M*. *alternatus* within the galleries. Research has shown that *P*. *zhonghuajia* itself also has good control effects on wood-boring beetles, such as *Semanotus bifasciatus* larvae [42]. Hence, Releasing *D. helophoroid* adults with *P. zhonghuajia* is suitable for forest stands with high population density and severe damage caused by *M. alternatus*, which can rapidly reduce damage.

Using biological and non-biological media to carry *B*. *bassiana* can enhance pest control efficacy [22]. Burying wheat bran containing *B*. *bassiana* under the bark of host trees can increase infection rates [43]. Using bark beetles to carry *B*. *bassiana* spores can increase their infection rate in longicorn beetles [44]. Using *Scleroderma* sp. to carry *B*. *bassiana* can actively infect *M*. *alternatus*, significantly improving transmission efficiency [29,45]. Using *Trichogramma dendrolimi* carrying *B*. *bassiana* to infect *Ostrinia furnacalis* can significantly enhance the pest control efficacy compared to using *T*. *dendrolimi* alone [46]. The results of our experiment indicated that *D. helophoroid* adults carrying *B*. *bassiana* can significantly improve the control efficacy against *M*. *alternatus*, but the control efficacy was limited in the short term (60 days). We speculate that this may be related to indoor conditions that are unsuitable for the growth and spread of *B*. *bassiana*, or to differences in the carrying capacity and lethal concentration of different fungal strains [30]. Meanwhile, the field application of *B*. *bassiana* still faces many challenges, such as fungal spores being easily affected by temperature, ultraviolet light, humidity, and other factors under natural conditions, leading to unstable activity and poor control efficacy in forest environments [20]. Therefore, it is necessary to continuously develop *B*. *bassiana*, optimizing its quality and yield through fermentation processes and other measures to enhance its control efficacy. In the future, a combination of three biocontrol agents can also be used (carrying both *P*. *zhonghuajia* and *B*. *bassiana*), or multiple biological control targets can be combined on optimized drill holes.

The technical prospects for controlling *M*. *alternatus* using *D. helophoroid* as the primary method are broad. However, all the results of the synergism approach in this study were obtained indoors, while field applications are prone to interference from various biological and abiotic factors [47,48]. To achieve better control effects in practical applications, several aspects still need improvement, such as enhancing the efficiency of large-scale indoor breeding [49,50,51], optimizing field release techniques [35,52,53], and enhancing the parasitic capacity of natural enemies [33,54]. Coordinating the application of different control methods holds significant practical implications for enhancing the effectiveness of biological control.

## 5. Conclusions

This study systematically validated three synergistic strategies for controlling *M*. *alternatus* using *D*. *helophoroides*: optimization of release density, regulation of drill hole parameters in lure logs, and biological synergy. The results indicate that accurately releasing 50 eggs/m or 3 adults/m, optimizing the drilling location on the lure logs (2 cm above the fecal hole) and release density (2 holes/m), and employing a synergistic mode where *D*. *helophoroides* (3 adults/m) carries either *P*. *zhonghuajia* (0.6 million/m) or *B*. *bassiana* (3 g/m) significantly increased the *M*. *alternatus* mortality rate. Our work not only confirms the effectiveness of *D*. *helophoroides* as a biological control agent but also innovatively proposes a composite synergistic mechanism combining drilling optimization and natural enemy carrier synergy, providing new insights for the control of boring pests. Compared to single biological release strategies, the integrated approach in this study reduces tree damage while enhancing control efficacy, aligning with the sustainable development needs of ecological forestry. While this study expands the understanding of *D*. *helophoroides*-mediated synergy against *M*. *alternatus* under controlled conditions, further translation into practical biological control requires strategy optimization adapted to actual forest environments to improve field efficacy.

## Figures and Tables

**Table 1:** Effect of lure logs drilling hole numbers on parasitism and predation of *Monochamus alternatus* by *Dastarcus helophoroides.*

Number of Drilling Holes (Holes/m)	Total Number of *Monochamus alternatus* in Lure Logs	Total Number of Larvae and Adults of *Dastarcus helophoroides*	Total Number of Parasitized *Monochamus alternatus*	Total Predation of *Monochamus alternatus*	Mortality of *Monochamus alternatus* (%)
1	157	56	41	4	28.7 ± 2.8 b
2	162	67	54	4	35.8 ± 2.3 a
3	154	53	50	6	36.4 ± 1.0 a

Data in the table refer to mean ± SD. The different lowercase letters following the data indicated significant differences in the mortality rate of *M. alternatus* at different drilling hole numbers 150 days after the release of *D. helophoroides* adults (*p* < 0.05).

**Table 2:** Effect of *Monochamus alternatus* by releasing *Dastarcus helophoroides* adults carrying *Pyemotes zhonghuajia.*

Release Density (Million Individuals/m)	Mortality of *Monochamus alternatus* (%)	
	*Pyemotes zhonghuajia* Carried by *Dastarcus helophoroides*	*Pyemotes zhonghuajia* Separate Release
0.2	29.7 ± 1.8 aBC	15.5 ± 1.4 bBC
0.4	48.2 ± 2.7 aAB	24.5 ± 2.7 bAB
0.6	67.6 ± 1.1 aAB	30.4 ± 1.0 bAB

Data in the table refer to mean ± SD. The different lowercase letters following the data indicate that, under the same *P. zhonghuajia* inoculation ratio, there was a significant difference in the mortality rate of *M. alternatus* between the group of *D. helophoroides* adults carrying *P. zhonghuajia* and the group where *P. zhonghuajia* were released alone after 60 days of treatment (*p* < 0.05); different uppercase letters indicate significant differences in the mortality rate of *M. alternatus* after 60 days of treatment at different inoculation ratios of *P. zhonghuajia* (*p* < 0.05). There were no dead individuals in the blank control group.

**Table 3:** Effect of *Monochamus alternatus* by releasing *Dastarcus helophoroides* adults carrying *Beauveria bassiana*.

Release Density (g/m)	Mortality of *Monochamus alternatus* (%)	
	*Beauveria bassiana* Carried by *Dastarcus helophoroides*	*Beauveria bassiana* Separate Spray
1	12.0 ± 1.5 a	5.2 ± 1.3 b
2	11.1 ± 2.5 a	7.1 ± 2.4 b
3	15.2 ± 1.3 a	8.1 ± 1.6 b

Data in the table refer to mean ± SD. The different lowercase letters following the data indicated that, under the same proportion of *B. bassiana* spray application, there was a significant difference in the mortality rate of *M. alternatus* between the group of *D. helophoroides* adults carrying *B. bassiana* and the group sprayed with *B. bassiana* alone after 60 days of treatment (*p* < 0.05). There were no dead individuals in the blank control group.

## Data Availability

The original contributions presented in this study are included in the article/Supplementary Materials. Further inquiries can be directed to the corresponding author.

## Supplementary Materials

The following supporting information can be downloaded at: https://www.mdpi.com/article/10.3390/insects16111138/s1, Table S1. Number and mortality rate of predation and parasitism of *M. alternatus* by *D. helophoroides.*

## Abbreviations

The following abbreviations are used in this manuscript:

PWN	Pine wood nematode (*Bursaphelenchus xylophilus*)
PWD	Pine wilt disease
